# Biotechnological strategies to improve production of microbial poly-(3-hydroxybutyrate): a review of recent research work

**DOI:** 10.1111/1751-7915.12129

**Published:** 2014-06-05

**Authors:** C Peña, T Castillo, A García, M Millán, D Segura

**Affiliations:** 1 Departamento de Ingeniería Celular y BiocatálisisMorelos, México; 2 Departamento de Microbiología Molecular, Instituto de Biotecnología, Universidad Nacional Autónoma de MéxicoMorelos, México

## Abstract

Poly-(3-hydroxybutyrate) [P(3HB)] is a polyester synthesized as a carbon and energy reserve material by a wide number of bacteria. This polymer is characterized by its thermo-plastic properties similar to plastics derived from petrochemical industry, such as polyethylene and polypropylene. Furthermore, P(3HB) is an inert, biocompatible and biodegradable material which has been proposed for several uses in medical and biomedical areas. Currently, only few bacterial species such as *Cupriavidus necator*, *Azohydromonas lata* and recombinant *Escherichia coli* have been successfully used for P(3HB) production at industrial level. Nevertheless, in recent years, several fermentation strategies using other microbial models such as *Azotobacter vinelandii, A. chroococcum*, as well as some methane-utilizing species, have been developed in order to improve the P(3HB) production and also its mean molecular weight.

## Introduction

Poly-(3-hydroxybutyrate) [P(3HB)] is produced and intracellularly accumulated as a carbon and energy reserve material. It can be produced by various bacteria, such as *Cupriavidus necator*, several species of *Pseudomonas, Bacillus, Azotobacter* and also recombinant *Escherichia coli*, expressing the P(3HB) biosynthetic genes from *C. necator* and *A. vinelandii* (Centeno-Leija *et al*., 2014). Since its discovery, P(3HB) has been used as substitute for bulk plastics, such as polyethylene and polypropylene, in the chemical industry. More recently, and based on its properties of biocompatibility and biodegradability, new attractive applications for P(3HB) have been proposed in the medical and pharmaceutical fields, where chemical composition and product purity are critical (Williams and Martin, 2005). In the medical field, P(3HB) has been used in artificial organ construction, drug delivery, tissue repair and nutritional/ therapeutic uses (Chen and Wang, 2013).

In all these applications, the molecular mass of P(3HB) is a very important feature to consider, because this determines the mechanical properties of the polymer, and in turn, the final applications. From a biotechnological point of view, the manipulation of the molecular mass of P(3HB) by means of the use of new strains and manipulating the culture conditions, seems to be a convenient method that could considerably improve the properties of P(3HB), expanding the potential application of this polymer, especially in the medical field.

Poly-(3-hydroxybutyrate) is produced by fermentation, either in batch, fed batch or continuous cultures using improved bacterial strains, cultured on inexpensive carbon sources such as beet and cane molasses, corn starch, alcohols and vegetable oils, combined with multi-stage fermentation systems (Lee, 1996; Chen and Page, 1997; Chen, 2009; 2010; Chanprateep, 2010; Peña *et al*., 2011). All these strategies have been attempted to improve both the yields and process productivity in order to have a more competitive process.

There are several reviews regarding the properties and applications of P(3HB); as well as about the different microorganisms producing P(3HB) (Byrom, 1987; Sudesh *et al*., 2000; Chen, 2009; 2010; Grage *et al*., 2009; Chanprateep, 2010; Peña *et al*., 2011); however, there are not recent reviews about the fermentation strategies for improving the P(3HB) production.

This review aims to summarize the recent trends in the bacterial production of P(3HB) using novel fermentation strategies combined with the use of genetic engineering to improve productivity and quality (in terms of its molecular weight) of P(3HB) that could be applied for its commercial production.

## P(3HB): structure and properties

Polyhydroxyalkanoates (PHAs) are linear polyesters conformed by hydroxyacyl units. They can be found as homopolymers or as copolymers containing combined 2-, 3-, 4-, 5- or 6-hydroxyacids (Sudesh *et al*., 2000; Kessler and Witholt, 2001; Chen, 2010). Polyhydroxyalkanoates classification depends on the number of carbon atoms present in their monomers as short-chain-length PHAs (scl-PHA; three to five C-atoms) and medium-chain-length PHAs (mcl-PHA; with six or more C-atoms) (Pan and Inoue, 2009).

**Fig. 1:**
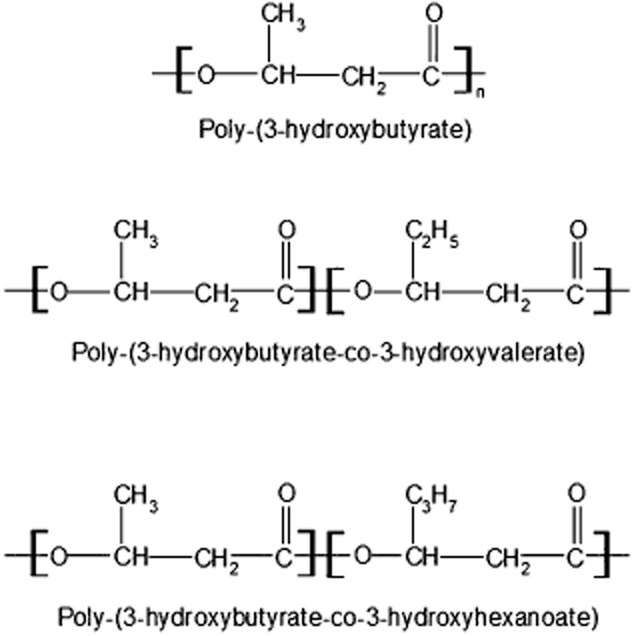
Chemical structure of poly-(3-hydroxybutyrate), poly-(3-hydroxybutyrate-co-3-hydroxyvalerate) and poly-(3-hydroxybutyrate-co-3-hydroxyhexanoate).

Interest in these polymers has increased in the last decades due to their thermoplastic properties, which make them a biodegradable and environmentally friendly alternative to petroleum based plastics, such as polyethylene and polypropylene. Although PHAs include a broad number of polymers of diverse monomeric composition, only few of them have been incorporated into the large-scale production: P(3HB); poly-(3-hydroxybutyrate-co-3-hydroxyvalerate) [P(3HB-co-3HV)] and poly-(3-hydroxybutyrate-co-3-hydroxyhexanoate) [P(3HB-co-3HHx)] (Chen, 2009; Chanprateep, 2010; Fig. 1).

Poly-(3-hydroxybutyrate) is the homopolymer of (R)-3-hydroxybutyrate units. It can be obtained within a wide range of molecular masses fluctuating from 200 to up to 20 000 KDa (Kusaka *et al*., 1998; Sudesh *et al*., 2000). The thermoplastic properties of P(3HB) and its biodegradability, without generation of toxic by-products, make it a sustainable alternative to petroleum-based plastics. In addition, this polymer is produced by biotechnological strategies allowing the control of its chemical composition, and therefore its physicochemical properties. Besides, this polymer shows interesting properties such as a high biocompatibility with mammalian cells, making them suitable for medical applications (Chen, 2009; 2010; Grage *et al*., 2009; Pan and Inoue, 2009; Shishatskaya *et al*., 2011; Bornatsev *et al*., 2013).

P(3HB) is a semi-crystalline polymer, characterized by a polymorphic crystallization, that is able to crystallize into two forms, α and β (Pan and Inoue, 2009). The α-form which consists in lamellar crystals, being the most common conformation for P(3HB) crystals (Pan and Inoue, 2009; Kabe *et al*., 2012) and the β-form characterized as a planar zigzag conformation which has been reported in films and fibres with high tensile strength (Iwata, 2005; Pan and Inoue, 2009; Kabe *et al*., 2012). It must be emphasized that the crystallization process affects the thermal and mechanical properties, as well as biodegradability of biopolymers (Pan and Inoue, 2009).

The thermoplastic and crystallization properties of P(3HB) are highly dependent of its molecular mass. Poly-(3-hydroxybutyrates) of low molecular masses (< 1 × 10^3^ kDa) are characterized by their brittleness and an early thermal degradation, near their melting temperature (above 180°C) (Hong *et al*., 2013). This behaviour has been explained as a result of its α-form crystallization (Kabe *et al*., 2012); however, increasing P(3HB) molecular mass improves the mechanical properties of films and fibres by promoting the β-form crystallization (Kabe *et al*., 2012). In this line, using P(3HB) of ultra-high molecular weight (UHMW; *M_w_* = 5.3 × 10^3^ kDa), Iwata (2005) reported that the tensile strength of the polymer could be manipulated from 38 to 1320 MPa, only by modifying the drawing method. This last value (1320 MPa) is higher than the tensile strength reported for polyethylene, polypropylene, polyvinyl alcohol and polyglycolic acid used at industrial level (Iwata, 2005).

However, up to now the UHMW-P(3HB) production has been restricted only for cultivations of low cell density, such as the cultures of recombinant *E. coli* XL-1 Blue (pSLY105), harbouring the *Cupriavidus necator* P(3HB) biosynthetic genes *phbCAB* (Kusaka *et al*., 1998; Iwata, 2005; Murakami *et al*., 2014; Kabe *et al*., 2012), mixed cultures of methane-utilizing bacteria (Helm *et al*., 2008) and *Azotobacter* cultivations (Peña *et al*., 2014). Therefore, several strategies have been designed to improve the thermo-mechanical properties of P(3HB) including: P(3HB) composites with other PHAs [P(3HV) or P(3HHx)] or other biopolymers (i.e.: cellulose, chitosan; Rajan *et al*., 2012), the addition of chemical plasticizers (i.e.: polyethylene glycol, glycerol, glycerol triacetate, 4-nonylphenol; Hong *et al*., 2013), as well as the blending of P(3HB) of different molecular masses (Kabe *et al*., 2012; Hong *et al*., 2013).

As shown in Table 1, it is possible to modify and improve the thermo-mechanical properties of P(3HB) for specific applications by combining P(3HB) of medium molecular weight with UHMW-P(3HB) (Sharma *et al*., 2004; Kabe *et al*., 2012) or P(3HB) of very low molecular weight [LMW-P(3HB); *M_w_* = 1.76 kDa] (Hong *et al*., 2013). In this line, blending P(3HB) of medium molecular weight with only 5% of UHMW-P(3HB) increased the tensile strength and elongation at break up to 33% and 48%, reaching values similar to those of conventional plastic films (Kabe *et al*., 2012). In contrast, addition of LMW-P(3HB) reduces polymer crystallinity, as well as the melting and crystallization temperature of P(3HB), but positively affects elongation at break and degradation rate (Hong *et al*., 2013), being this last characteristic of great interest for biomedical applications.

**Table 1:** Thermo-mechanical properties of P(3HB) and its composites with UHMW-P(3HB) or LMW-P(3HB)

Compound	Drawn ratio	*T_g_* (°C)	*T_c_* (°C)	*T_m_* (°C)	Tensile strength (MPa)	Elongation at break (%)	Young's modulus (GPa)	Crystallinity (%)	Reference
P3HB	12^a^	1.8	53	170	161	45	2.8	78	Kabe *et al*., 2012
UHMW	10^a^	2.4	57	172	191	56	1.6	73	Kabe *et al*., 2012
UHMW/P3HB (5/95)	12^a^	2.2	53	170	242	88	1.5	75	Kabe *et al*., 2012
UHMW	60^b^	n.d.	n.d.	n.d.	1320	35	18.1	n.d.	Iwata, 2005
P3HB/LMW (87.5/12.5)	None	−2.6	93	162.3	23.4	4.2	n.d.	44.8	Hong *et al*., 2013
P3HB/LMW (83.3/16.6)	None	−4.8	82	160.5	24.3	9.8	n.d.	40.4	Hong *et al*., 2013
P3HB/LMW (75/25)	None	−7.3	76	155.8	11.6	3.8	n.d.	37.8	Hong *et al*., 2013

a. Processed by cold drawing.

b. Processed by cold drawing/two step drawing.

T_g_, temperature to glass transition; T_c_, crystallization temperature; T_m_, melting temperature; n.d., not described.

## Biomedical applications of P(3HB)

Previous reviews have focused on novel applications of P(3HB) and other PHAs in several biomedical areas (Chen and Wu, 2005; Chen, 2009; Grage *et al*., 2009; Peña *et al*., 2011; Chen and Wang, 2013) which can be described as follows: material for sutures and tissue engineering, including heart valves, bone scaffolding, scaffolds for skeletal myotubes and nerve tissue (Grage *et al*., 2009; Ricotti *et al*., 2012; Masaeli *et al*., 2013); nano or micro beads for drug delivery and target-specific therapy for treatment of illness such as cancer and tuberculosis (Grage *et al*., 2009; Parlane *et al*., 2012; Althuri *et al*., 2013); and finally, its possible application as biomarker or biosensor (Grage *et al*., 2009). Table 2 summarizes some of the more recent attempts to apply P(3HB) in these fields, mainly as tissue engineering scaffolds and micro or nanoparticles for drugs delivery. It must be emphasized that, for these applications, P(3HB)s of a wide range of molecular weights (MW) have been used. For applications such as nano- or microparticles, the MW did not affect the production yield of particles (Shishatskaya *et al*., 2011). On the other hand, P(3HB) used for tissue engineering, in some cases requires to be mixed with materials such as chitosan (Cao *et al*., 2005; Medvecky *et al*., 2014; Mendonca *et al*., 2013), other PHAs (Masaeli *et al*., 2013), polyethylene glycol (PEG) (Chan *et al*., 2014), hydroxyapatite (Shishatskaya *et al*., 2006; Ramier *et al*., 2014) or even cell growth inductors (Filho *et al*., 2013). Addition of those materials allows to improve not only the mechanical properties of P(3HB) but also its degradability, hydrophilicity and its cell attachment capabilities.

**Table 2:** Biomedical applications of P(3HB) with different molecular weights

	Applications	P(3HB) MW (kDa)	Preparation procedure	Reference
P(3HB) LMW	Osteoblast scaffolds	220	P3HB and hydroxyapatite were mixed using mechanical and physical methods	Shishatskaya *et al*., 2006
	Scaffolds	89–110	Blends of P3HB and chitosan at different ratios were evaluated	Medvecky *et al*., 2014
	Nanofibrous scaffolds for bone tissue engineering	144	Electrospinning/electrospraying, P3HB and hydroxyapatite	Ramier *et al*., 2014
P(3HB)	Nanoparticles for retinoic acid (RA) delivery	350	50 nm particles of P3HB/RA were prepared by dialysis	Errico *et al*., 2009
	Microcapsules for drugs delivery	300	Microcapsules of 0.5–1.5 μm with P3HB and smectite clays were formed	da Silva-Valenzuela *et al*., 2010
	Scaffolds of PHB and otholits (osteoinductor) for bone tissue regeneration	300	Solutions of P3HB and otholits (1% w/w) were electrospinning	Filho *et al*., 2013
	Scaffolds 3D for osteoblasts engineering	524	P3HB and chitosan blends were evaluated	Mendonca *et al*., 2013
	Scaffolds for tissue engineering	300	P3HB scaffolds were prepared by salt leaching and electrospinning	Masaeli *et al*., 2012
	Nanofibrous scaffolds nerve tissue engineering	437	Blends of P3HB (50)/PHBV (50) were treated by electrospinning	Masaeli *et al*., 2013
P(3HB) UHMW	Scaffolds for tissue engineering	890	Chitosan and P3HB films were prepared by emulsion blending	Cao *et al*., 2005
	Scaffolds for nerve cells	1143	P3HB was treated with PEG reducing 10 fold-times its MW but promote cell growth	Chan *et al*., 2014

## Producers of P(3HB)

**Fig. 2:**
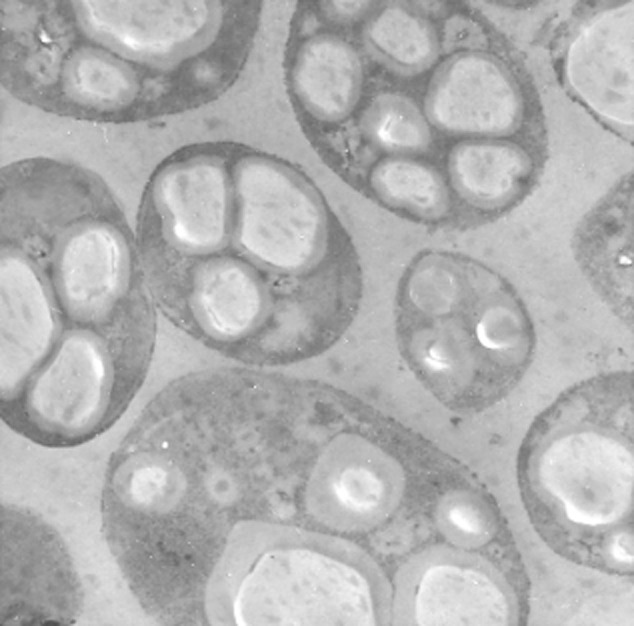
Transmission electron micrograph of a thin section of *A. vinelandii* containing P(3HB) granules (white inclusions).

The ability to synthesize and accumulate P(3HB) and other PHAs as a carbon and energy reserve material is widespread among the prokaryotes. More than 300 species, mainly of bacteria, have been reported to produce these polymers (Olivera *et al*., 2001; Chanprateep, 2010). However, not all of these microorganisms have been shown to accumulate sufficient P(3HB) for large-scale production. Among the bacteria that are able to accumulate large amounts of PHA are *C. necator* (formerly known as *Ralstonia eutropha* or *Alcaligenes eutrophus*), *Azohydromonas lata* (also known as *Alcaligenes latus*), *Pseudomonas oleovorans*, *Pseudomonas putida, Aeromonas hydrophila*, *Paracoccus denitrificans*, *Methylobacterium extorquens*, *Bacillus spp*., *Azotobacter vinelandii* and recombinant *E. coli*, expressing the P(3HB) biosynthetic genes from *C. necator, A. lata* or *A. vinelandii* (Lee, 1996; Olivera *et al*., 2001; Chen, 2009; Centeno-Leija *et al*., 2014). Figure 2 shows *A. vinelandii* cells with granules of P(3HB). From the microorganisms mentioned, the more successful species for production at pilot or large scale are *C. necator*, *A. lata* and recombinant *E. coli*, being able to accumulate up to 80% of the polymer from a final dry cell weight of up to 200, 60 and 150 g l^−1^ respectively (Chen, 2009).

Many species of Archaea have also been shown to be PHA producers, particularly members of *Haloarchaea* (Legat *et al*., 2010; Poli *et al*., 2011). These organisms could present important advantages as PHA producers because they can utilize cheap carbon sources (Huang *et al*., 2006), they do not need strict sterilization (they are able to grow in hypersaline conditions, in which very few organisms can survive), and because they can release the polymer produced easily because they lyse in distilled water, facilitating its isolation and lowering the production costs (Hezayen *et al*., 2000; Poli *et al*., 2011). The carbohydrate-utilizing species *Haloferax mediterranei* is particularly interesting because it accumulates large amounts of P(3HB) on glucose or starch, it grows optimally with 25% (w/v) salts and accumulates 60–65% of polymer (w/w) (Rodriguez-Valera and Lillo, 1992). *H. mediterranei*, shows the highest potential for industrial application because it can reach cell concentrations of 140 g l^−1^, with a PHA content of 55.6% reaching a PHA concentration of 77.8 g l^−1^ in a repeated fed-batch fermentation (Huang *et al*., 2006), and it is also able to produce a P(3HB-co-P3HV) copolymer (10.4 mol% 3HV) from enzymatic extruded starch (Chen *et al*., 2006).

## Metabolic pathways and genetics involved in production of P(3HB)

**Fig. 3:**
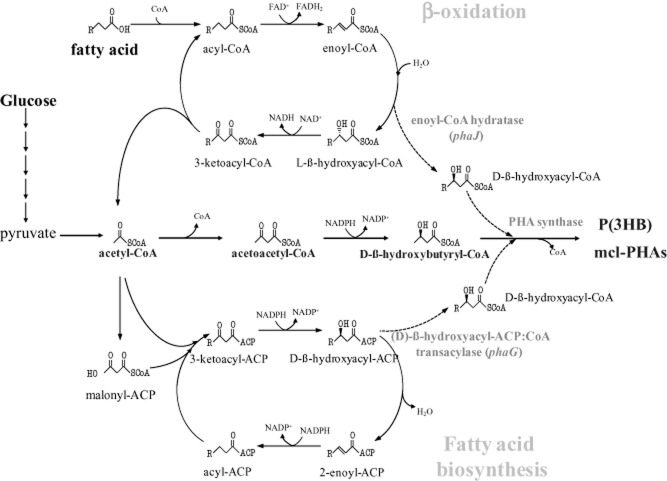
Metabolic pathways and genetics involved in the production of P(3HB).

The biosynthetic pathway for P(3HB) (Fig. 3) starts with the condensation of two molecules of acetyl-CoA to form acetoacetyl-CoA. The enzyme catalyzing this reaction is 3-ketothiolase, encoded by the *phbA* gene. An acetoacetyl-CoA reductase (gene *phbB*) coverts the acetoacetyl-CoA to 3-hydroxybutyryl-CoA using NADPH. Finally, the enzyme PHA synthase (encoded by *phbC*) polymerizes the 3-hydroxybutyryl-CoA monomers to P(3HB), liberating CoA (Rehm, 2003; Stubbe *et al*., 2005) (Fig. 3). In some species, the P(3HB) biosynthetic genes *phbA*, *phbB* and *phbC* are clustered and are presumably organized in one operon *phbCAB* (Reddy *et al*., 2003); although this gene order varies from species to species, and the genes can also be unlinked. More than 60 PHA synthase genes (*phbC* or *phaC*) from eubacteria have been cloned and sequenced, and many more have been revealed in the bacterial genomes sequenced (Steinbüchel and Lütke-Eversloh, 2003). Other genes whose products are also involved in PHA metabolism and their specific metabolic roles have been reviewed by Chen (2010).

Besides P(3HB), other PHAs containing 150 different monomers have been reported. This PHA diversity is due to the broad substrate range exhibited by the PHA synthases, the PHA polymerizing enzymes (Steinbüchel and Lütke-Eversloh, 2003; Stubbe *et al*., 2005; Volova *et al*., 2013). The different PHAs are synthesized depending also on the carbon source provided; the metabolic routes present to convert that carbon source into the hydroxyacyl-CoA monomers, and the specificity of the PHA synthase of that particular organism. The biosynthetic pathways reported up to date have been reviewed recently (Lu *et al*., 2009; Chen, 2010; Panchal *et al*., 2013), so we only present a brief description of the routes involved. For the synthesis of PHAs composed of 3-hydroxyalkanoic acids of C6–C16 (referred to as mcl-PHAs) the hydroxyacyl-CoA precursors are derived from fatty acid metabolism (Fig. 3). These precursors can be obtained either from ß-oxidation of alkanes, alkanols or alkanoic acids (De Smet *et al*., 1983; Brandl *et al*., 1988; Lagaveen *et al*., 1988), mainly by an enantioselective enoyl-CoA hydratase (encoded by *phaJ*) that produces the (R)-hydroxyacyl-CoA (Tsuge *et al*., 2003), or from fatty acid *de novo* biosynthesis using an (R)-3-hydroxyacyl-ACP:CoA transacylase (encoded by *phaG*) to produce the substrates for the PHA synthase from a non-related carbon source, such as carbohydrates (Rehm *et al*., 1998; Hoffmann *et al*., 2000a,b; Matsumoto *et al*., 2001).

## Molecular strategies to improve P(3HB) production

Although many of the P(3HB) production systems use non-genetically modified bacterial strains, some efforts have been undertaken to increase the production of these polymers by genetic manipulation. These efforts include mainly the modification of the metabolism to favour P(3HB) synthesis, the modification of regulatory systems controlling P(3HB) synthesis and recombinant *phb* gene expression.

The P(3HB) biosynthetic routes compete for precursors with central metabolic pathways, such as the tricarboxylic acid (TCA) cycle, fatty acid degradation (ß-oxidation) and fatty acid biosynthesis. They also compete with other biosynthetic pathways that use common precursors. Three examples of genetic modifications that favour P(3HB) synthesis by metabolism modification of the producer strain were reported in *A. vinelandii*. Page and Knosp (1993) reported a strain (UWD), which has a mutation in the respiratory NADH oxidase that resulted in the ability to accumulate P(3HB) during the exponential phase without the need of nutrient limitation. The second example is found in the inactivation of pyruvate carboxylase, the anaplerotic enzyme catalyzing the ATP-dependent carboxylation of pyruvate, to generate oxaloacetate that replenishes the TCA cycle (Segura and Espín, 2004). This mutation increased three times the specific production of P(3HB) (g_P(3HB)_ g_protein_^-1^), in contrast with the wild type strain *A. vinelandii* UW136, probably as a result of a diminished flux of acetyl-CoA into TCA cycle, leaving it available for P(3HB) synthesis. In the same bacterium, a mutation blocking the synthesis of alginate, an exopolysaccharide produced by this organism, increased the P(3HB)-specific production up to five times, depending on the growth conditions evaluated, with a higher yield based on glucose as compared with the wild type strain ATCC9046. The mutation not only increased the capacity of the bacterium to produce P(3HB) per biomass unit, but also allowed an increased growth, raising the volumetric production of the polymer up to 10-fold (Segura *et al*., 2003).

**Fig. 4:**
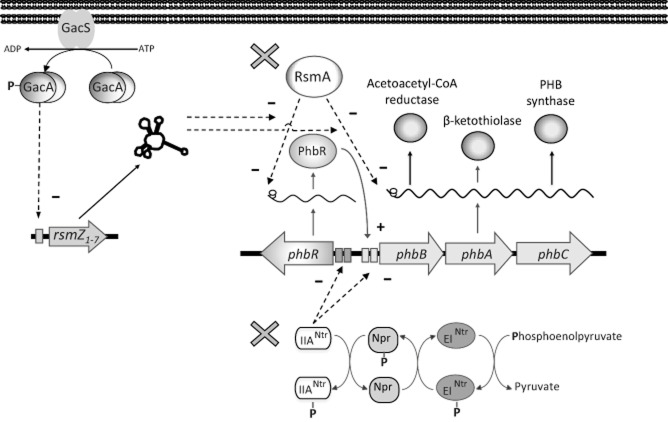
Model of the regulatory systems controlling the expression of the *phb* genes in *A. vinelandii*. (+) indicate positive regulation; (−) indicate negative regulation. Promoters are indicated as rectangles. The regulators inactivated in the *A. vinelandii* improved strains OPN and OPNA are indicated by a grey cross.

Regarding the modification of regulatory systems controlling PHA synthesis to increase their production, some interesting examples are also found in *A. vinelandii*. Poly-(3-hydroxybutyrate) synthesis in this bacterium is regulated by the nitrogen-related phosphotransferase system (PTS^Ntr^), where the non-phosphorylated form of the IIA^Ntr^ protein negatively regulates the expression of the P(3HB) biosynthetic operon (Segura and Espín, 1998; Noguez *et al*., 2008). Another system regulating P(3HB) synthesis in *A. vinelandii* is the post-transcriptional regulatory system RsmZ/Y-A, where the RsmA protein represses translation of the mRNAs of the *phbBAC* biosynthetic operon and of *phbR* that codes for its transcriptional activator (Hernández-Eligio *et al*., 2012). In each case, negative regulators IIA^Ntr^ and RsmA were identified (Fig. 4). In order to have P(3HB) overproducing strains of *A. vinelandii* OP, the gene coding for the IIA^Ntr^ (*ptsN*) was inactivated. This mutation increased 77% the specific production of P(3HB), equivalent to 4.1 g l^−1^ of PHB (3.5 g l^−1^ in the case of the wild type), with a 36% higher yield of product based on the consumed substrate (Peña *et al*., 2014). Later, a mutant where both negative regulators (IIA^Ntr^ and RsmA) were inactivated was constructed (Fig. 4), further increasing the P(3HB) accumulation capacity of *A. vinelandii*. This strategy, together with the implementation of a fermentation strategy allowed to produce 27 g l^−1^ of P(3HB) (García *et al*., 2013).

Another case illustrating production improvement by manipulation of regulatory systems is found in the cianobacterium *Synechocystis* sp. PCC 6803. In this bacterium, the overexpression of the sigma factor SigE, previously shown to activate the expression of many sugar catabolic genes and to enhance the levels of acetyl-CoA, increased the production of P(3HB) two or three times (Osanai *et al*., 2013).

Fast growth on simple media and the possibility to reach a high cell density in the culture with a high-content P(3HB) are important factors to consider for a successful P(3HB) production process. Because *E. coli* is an extensively studied bacterium with well-established technologies for genome manipulation, cultivation and downstream processing, many studies have focused on the use of *E. coli* to efficiently produce these polymers. This bacterium is a non-PHA producer; however, the genes of the P(3HB) producer *C. necator* H16 were cloned in *E. coli* for the first time by Slater *et al*^1988^. in 1988, enabling the production of P(3HB) in this organism. Since then, many different genetic modifications have been attempted, both to improve the accumulation of P(3HB) at low-cost with high productivity and to produce diverse copolymers using metabolic engineering and synthetic biology strategies. These strategies have been reviewed recently (Li *et al*., 2007; Wang *et al*., 2013).

## Fermentation strategies to improve the production of P(3HB)

### Effect of carbon source on P(3HB) production

The mayor expenses in the production of P(3HB) are determined by the cost of the fermentation substrate, the polymer extraction from the cells and the treatment of fermentation and extraction wastes (Chen, 2010). Of all these factors, the cost of the carbon source has the greatest influence on the price of P(3HB). Because of the above, new alternatives have been proposed to reduce the costs of raw materials. It is important to note that the selection of carbon sources should not focus only on the market prices but also on the availability and on global price (Chanprateep, 2010).

Table 3 summarizes different carbon sources used for the P(3HB) production. Fortunately, most P(3HB) producers can metabolize a wide range of raw materials. For example, it is known that several species of *Azotobacter* can use corn syrup, cane molasses, beet molasses or malt extract as carbon sources (Kim, 2000; Myshkina *et al*., 2008; Peña *et al*., 2011). For example, Kim (2000) reported the use of two inexpensive substrates, starch and whey, to produce P(3HB) in fed-batch cultures of *A. chroococcum* H23 and recombinant *E. coli*. These authors found that in fed-batch culture of *A. chroococcum* H23 a cell concentration of 54 g l^−1^ with 46% (w/w) P(3HB) was obtained with oxygen limitation, whereas 71 g l^−1^ of cells with 20% (w/w) P(3HB) was achieved without oxygen limitation. In the case of whey as carbon source, using recombinant *E. coli* 6576, Kim (2000) reported a P(3HB) content of 80%, with a cell concentration of 31 g l^−1^.

**Table 3:** Comparison of P(3HB) volumetric production, content and yields using different carbon sources

Organism	Carbon source	Quantity of carbon source (g) employed	DCW (g l^−1^)	P(3HB) concentration (g l^−1^)	P(3HB) content (%)	P(3HB) yield based on carbon source (g g^−1^)	Reference
*A. lata*	Sucrose	72.9	10.78	5.25	48	n.d.	Zafar *et al*., 2012a
*C. necator DSM545*	Glucose	523	164	125	76.2	0.22	Mozumder *et al*., 2014
	Waste glycerol	n.d	104.7	65.6	62.7	0.52	Mozumder *et al*., 2014
	Waste glycerol	170.8	76.2	38.1	50	0.34	Cavalheiro *et al*., 2009
	Pure glycerol	249	82.5	51.2	62	0.36	Cavalheiro *et al*., 2009
*A. chrococcum* H23	Alpechin/acetate	30/0.06	7.36	6.10	82.9	n.d	Pozo *et al*., 2002
	Starch	200	54	25	46	n.d	Kim, 2000
*E. coli recombinant GCSC 6576*	Whey	340	31	25	80	n.d.	Kim, 2000

n.d., data not described.

On the other hand, P(3HB) and P[3HB-co-3HV]) copolymers were produced by *A. chroococcum* strain H23, when growing in culture media supplemented with wastewater from olive oil mills (alphechin), as the sole carbon source (Pozo *et al*., 2002). A maximal concentration of P(3HB) of 6.2 g l^−1^ was reached when the cells were cultured in shaken flasks at 250 r.p.m. for 48 h at 30°C in liquid medium supplemented with 60% (v/v) alpechin and 0.12% (v/v) ammonium acetate (Table 3). Production of PHAs by *A. chroococcum* strain H23 using alpechin looks promising, as the use of a cheap substrate for the production of these materials is essential if bioplastics are to become competitive products.

In this context, crude glycerol (a by-product of the large-scale production of diesel oil from rape) has been evaluated for its potential use as a cheap feedstock for P(3HB) production (Cavalheiro *et al*., 2009; Mozumder *et al*., 2014). Bacteria used has been *C. necator* DSM 545, which accumulated P(3HB) from pure glycerol up to a content of 62.5% (w/w) of cell dry mass, reaching a volumetric production of 51.2 g l^−1^, with a yield on glycerol of 0.36 g _P(3HB)_ g _gly_^-1^ (Cavalheiro *et al*., 2009). On the other hand, when this by-product was used by Mozumder and colleagues (2014), a maximal biomass concentration of 104.7 g l^−1^ was reached, with a P(3HB) concentration in the broth culture of 65.6 g l^−1^. In addition, the molecular weight of P(3HB) produced with *C. necator* from glycerol varies between 7.86 × 10^2^ kDa (with waste glycerol) and 9.57 × 10^2^ kDa (with pure glycerol), which allows the processing by common techniques of the polymer industry (Cavalheiro *et al*., 2009).

A recent report on P(3HB) production using *A. lata* has been published (Zafar *et al*., 2012a). In this study, the optimization of P(3HB) production process using *A. lata* MTCC 2311 was carried out. By using a genetic algorithm on an artificial neural network, the predicted maximum P(3HB) production of 5.95 g l^−1^ was found, using 35.2 g l^−1^ of sucrose and 1.58 g l^−1^ of urea (Zafar *et al*., 2012a); however, the highest experimental P(3HB) concentration (5.25 g l^−1^) was achieved using 36.48 g l^−1^ of sucrose. The same authors reported that the use of propionic acid together with cane molasses allowed the synthesis of the copolymer P(3HB-co-3HV) in maximal concentrations of 7.2 g l^−1^ in shaken flasks and of 6.7 g l^−1^ in 3-L bioreactor (Zafar *et al*., 2012b).

### Fermentation strategies

Only a few species of bacteria producing P(3HB) have been used at industrial scale to produce the polymer. These include *C. necator*, *A. lata* and recombinant *E. coli* (Khanna and Srivastava, 2005). On the other hand, there are some bacteria, such as *A. vinelandii* and *A. chroococcum* which can accumulate a high P(3HB) content and therefore could be used for the synthesis of this polymer at large scale.

Several studies have been carried out which described the P(3HB) production by several microbial strains, either in batch, fed batch or continuous cultures. Batch fermentation for P(3HB) production is a popular process due to its flexibility and low operation costs. However, batch cultures have the disadvantage that, usually, the yields and productivities of P(3HB) are low. In this sense, the P(3HB) production in batch cultures of *C. necator* ATCC 17699 has been studied using acetic acid as a carbon source (Wang and Yu, 2001). In this study, the P(3HB) productivity was of only 0.046 g l^−1^ h^−1^ employing a carbon/nitrogen (C/N) weight ratio of 76, with a maximal accumulation of P(3HB) close to 50% (w/w).

The systems more often employed for P(3HB) production are those involving two or three stages. These fermentations have been widely used for the production of P(3HB) and other PHAs (Ruan *et al*., 2003; Rocha *et al*., 2008). The fed-batch cultures have been employed to achieve high cell densities and a high concentration of P(3HB) (Kulpreecha *et al*., 2009). Fed-batch cultivations are systems where one or more nutrients are supplied to the bioreactor and the products and other components are kept within the system until the end of fermentation. This means that there is an inflow but no outflow, and the volume changes with respect to time (Mejía *et al*., 2010). There are several ways to feed the cultures, and it is possible to add one or more components.

Currently there are reports in the literature about the use of exponentially fed-batch cultures for P(3HB) production with microorganisms as *A. lata* (Grothe and Chisti, 2000). These authors obtained a maximal biomass concentration of 36 g l^−1^ with a P(3HB) volumetric production of 20 g l^−1^ by varying the components in the culture. More recent studies have shown that a total concentration of 4.5 g l^−1^ of P(3HB) was obtained using limiting conditions of dissolved oxygen with processed cheese whey supplemented with ammonium sulfate in fed-batch culture *of Methylobacterium sp. ZP24* (Nath *et al*., 2008). This investigation reflects the possibility of developing a cheap biological route for production of green thermoplastics.

Recently, an integrated model was used for the optimization of the production of P(3HB) with tailor-made molecular properties in *A. lata*. A single-shot feeding strategy with fresh medium free of nitrogen was designed and experimentally tested. Using this strategy, a maximal concentration of P(3HB) of 11.84 g l^−1^ was obtained, equivalent to polymer content equal to 95% (w/w) of dry cell weight (Penloglou *et al*., 2012a).

Table 4 shows the more recent results reported about of maximal concentration and productivity of P(3HB) reached using different microorganism and fed-batch systems. From these studies, the cases for P(3HB) production using *Bacillus megaterium, C. necator*, recombinant *E. coli* and *Azotobacter* are highlighted. For example, Kulpreecha and colleagues (2009) reported a high P(3HB) production (30.5 g l^−1^) and P(3HB) productivity (1.27 g l^−1^ h^−1^) in a fed-batch culture of *B. megaterium* BA-019 using sugarcane molasses as a carbon source. More recently, Kanjanachumpol and colleagues (2013) found that in cultures of *B. megaterium* BA-019 with intermittent feeding of the sugarcane molasses and an increase of the C/N ratio at 12.5 improved the biomass and volumetric productivity of P(3HB), reaching a maximal biomass concentration of 90.7 g l^−1^ with 45.84% (w/w) of P(3HB) content and a productivity of 1.73 g l^−1^ h^−1^ P(3HB).

**Table 4:** Comparison of P(3HB) production using different microorganism and fed-batch strategies

Organism	Feeding strategy	DWC (g l^−1^)	P(3HB) (g l^−1^)	P(3HB) productivity (g l^1^ h^−1^)	P(3HB) content (% wt)	Reference
*B. megabacterium* BA-019	pH stat	72.6	30.5	1.27	42	Kulpreecha *et al*., 2009
	Intermittent	90.7	41.6	1.73	46	Kanjanachumpol *et al*., 2013
*C. necator*	Pulses	75	53	0.92	71	Tanadchangsaeng and Yu, 2012
	Pulses	83	67.2	2.5	81	Pradella *et al*., 2012
	Pulses	82.5	51.2	1.52	62	Cavalheiro *et al*., 2009
	Exponential + coupled to alkali addition monitoring + constant with N2 limitation	164	125	2.03	76.2	Mozumder *et al*., 2014
*E. coli*	pH stat	119.5	96.2	2.57	80	Ahn *et al*., 2000
*A. vinelandii*	Exponential + pulses	37.2	27.3	0.5	73.3	García *et al*., 2013

In the case of *C. necator*, Tanadchangsaeng and Yu (2012) reported a significant increase in P(3HB) volumetric production and productivity (53 g l^−1^ and 0.92 g l^−1^ h^−1^ respectively) in a fed batch using glycerol as a carbon source. Considering this, they suggested that glycerol is an ideal feedstock for producing bioplastics via bacterial fermentation due to its ubiquity, low price and high degree of reduction. However, the productivities reported using glycerol as carbon source (Cavalheiro *et al*., 2009) are still relatively low compared to other reports. An example is the high P(3HB) productivity reached by *C. necator*, using soybean oil in fed-batch culture (Pradella *et al*., 2012). In this study, the authors reported a maximal P(3HB) concentration of 67.2 g l^−1^ with a volumetric productivity of 2.5 g l^−1^ h^−1^. On the other hand, Mozumder and colleagues (2014) using *C. necator*, developed a three-stage feeding strategy using glucose as the sole carbon source that resulted in a P(3HB) concentration of 125 g l^−1^, with a P(3HB) content of 76% achieving a productivity of 2.03 g l^−1^ h^−1^.

Another successful case is that reported by Ahn and colleagues (2000), who developed fermentation strategies for P(3HB) production from whey by recombinant *E. coli* strain CGSC 4401. Using a pH stat fed-batch cultures, adding a concentrated whey solution containing 280 g l^−1^ was possible to reach final cell and P(3HB) concentrations of 119 and 96 g l^−1^ respectively, at 37.5 h, with a maximal productivity of 2.57 g l^−1^ h^−1^ (Table 4). The strategies developed in this study provide an attractive solution to whey disposal and utilization of this raw material for the P(3HB) production at large scale.

For several decades the synthesis of P(3HB) by *Azotobacter* strains has been the subject of studies, either in batch (Page and Knosp, 1993; Page *et al*., 2001; Myshkina *et al*., 2008), continuous (Senior *et al*., 1972; Senior and Dawes, 1973) or fed-batch cultures (Page and Cornish, 1993; Chen and Page, 1997; Kim and Chang, 1998; García *et al*., 2013). However, the information related with the fermentation systems has been scarce in recent years. On the other hand, to our knowledge, none of these processes has yet been adopted for the industrial production of P(3HB).

Recently, our group reported (García *et al*., 2013) a mixed fermentation strategy based on exponentially fed-batch cultures (EFBC) and nutrient pulses with sucrose and yeast extract to achieve a high concentration of P(3HB) by *A. vinelandii* OPNA, which carries a mutation on the genes encoding IIA^Ntr^ (*ptsN*) and RsmA (*rsmA*) that negatively regulate the synthesis of P(3HB). Using a strategy of exponential feeding coupled with nutrient pulses (with sucrose and yeast extract), the production of P(3HB) increased sevenfold (with respect to the values obtained in batch cultures) to reach a maximal P(3HB) concentration of 27.5 ± 3.2 g l^−1^ at 60 h of fermentation (Table 4). Overall, the use of the OPNA mutant of *A. vinelandii*, impaired in the P(3HB) regulatory systems, in combination with a mixed fermentation strategy, could be a feasible strategy to optimize the P(3HB) production at industrial level (García *et al*., 2013).

### Influence of the culture conditions on the P(3HB) molecular mass

The molecular mass (MM) of P(3HB) determines the elastic behaviour of the material and its mechanical resistance (Iwata, 2005). Fibres of P(3HB) with a MM of about 3.0 × 10^2^ kDa have a tensile strength of 190 MPa and an elongation at break of 5%. In contrast, the tensile strength of fibres of P(3HB)-UHMW with a MM of 5.3 × 10^3^ kDa could be manipulated to increase up to sevenfold (1320 MPa) with an elongation at break of 57% (Iwata, 2005). Therefore, for P(3HB) commercial production, it is desirable to obtain polymers with a suitable molecular mass for their final application, especially in the medical field.

It has been described by several authors how the P(3HB) molecular mass depends on the culture conditions such as: medium composition, pH and oxygen availability. In the next section, the influence of these parameters on the molecular weight of the P(3HB) will be discussed.

#### Medium composition

The effect of the medium composition on the P(3HB) MM has been reported for *Azotobacter* species, *C. necator, A. lata* and for methane-utilizing mixed cultures (Chen and Page, 1994; Wang and Yu, 2001; Helm *et al*., 2008; Myshkina *et al*., 2008; Penloglou *et al*., 2012b).

Wang and Yu (2001) reported that the mean molecular mass (MMM) of P(3HB) produced by *C. necator* could be altered by the medium composition, under chemically defined conditions and using acetic acid as carbon source. These authors evaluated the effect of C/N ratio on the MMM. The MMM of the polymer was higher (8.2 × 10^2^ kDa) in cultures developed under low C/N ratio, with respect to those obtained under high C/N ratio (MM = 5.2 × 10^2^ kDa) (Table 5). However, the amount of P(3HB) per residual biomass increased from 0.5 to 1.2 g _P(3HB)_ g _biomass_^-1^ increasing the C/N ratio.

**Table 5:** Influence of culture conditions on the molecular mass of PHB

Organism	Carbon Source	Condition	MMW (kDa)	PHB content (%)	Reference
*C. necator*	Acetic Acid	Low C/N ratio = 4	820	50	Wang and Yu. 2001
		High C/N ratio = 72	520		
*A. lata*	Sucrose	C/N ratio = 20	2576	15	Penloglou *et al*., 2012b
		C/N ratio = 8	596	35	
		C/P ratio = 8	2076	27	
*A. vinelandii UWD*	Beet molasses	5% (w/v)	4100	N.S.	Chen and Page, 1994
	Beet molasses	10% (w/v)	3500		
	Sucrose	5% (w/v)	1600		
*A. chroccoccum 7B*	Sucrose	2% (w/v)	1200–1600	74–79	Myshkina *et al*., 2008
	Sucrose+Molasses		590	60	
*E. coli XL-1*	Glucose	pH = 6.0–7.0	2000–2500	32–35	Bocanegra *et al*., 2013
	Xylose				
*A. chroccoccum 6B*	Glucose	0.5 VVM	1100	63.5	Quagliano and Miyazaki, 1997
		2.5 VVM	100	7.6	
*A. vinelandii OPN*	Sucrose	Low aeration	2020	67	Peña *et al*., 2014
		High aeration	1010	62	

N.S., not specified.

On the other hand, in *A. lata*, Penloglou and colleagues (2012b) evaluated in 2-L shaken flasks cultures the effect of the initial C/N ratio and carbon/phosphates (C/P) weight ratio on the MM of P(3HB). These authors reported that the polymer reached highest MMM values (2.5 × 10^3^ kDa) for a C/N ratio of 20 and an MM of 2.0 × 10^3^ kDa when C/P ratio was 8; however, under such growth conditions, the P(3HB) accumulation was lower than 30%. Also, these authors observed that the MM diminished up to 20 and 3 times-fold as the C/N or C/P ratios decreased to 6 and 0.8, respectively.

The role of the potassium, iron and sulfur deficiency on the MM of the P(3HB) has been studied in methane-utilizing mixed cultures by Helm and colleagues (2008). In two-stages cultures (with a continuous-growth phase and a discontinuous P(3HB)-accumulation phase), P(3HB) accumulation was higher in those cultures under potassium deficiency (33.6% w/w) than the accumulation obtained under iron and sulfur-deficiency conditions. With respect to the MM of the P(3HB), the highest value (3.1 × 10^3^ kDa) was obtained in the cultures developed under potassium deficiency, and the lowest value (1.7 × 10^3^ kDa) was achieved in those cultures lacking iron. It must be emphasized that the MM of 3.1 × 10^3^ kDa is up to now the highest value reported for methanotrophic bacteria.

In the case of *A. vinelandii*, Chen and Page (1994) reported that strain UWD produced a polymer with a high-molecular weight (4.1 × 10^3^ kDa), when this bacterium was grown in 5% w/v beet molasses medium. The polymer MM decreased when the beet molasses concentration was increased. Similar results were obtained in equivalent concentrations of sucrose (as raw sugar), but the polymer MM was not greater than 1.6 × 10^3^ kDa (Table 5).

For the producer strain *A. chroococcum* 7B, it has been shown that the MM of P(3HB) depends on changes in the medium composition, specially carbon source (Myshkina *et al*., 2008). These authors described that the MM of P(3HB) obtained using glucose, sucrose or starch as carbon sources, oscillated around 1.2 × 10^3^ and 1.6 × 10^3^ kDa (Table 5). However, the P(3HB) MM decreased to 5.9 × 10^2^ kDa when *A. chroococcum* 7B was cultured using sucrose complemented with molasses at 2% (w/v). The negative effect of the introduction of molasses suggested that presence of organic acids in this kind of raw material affected P(3HB) biosynthesis. To confirm this behaviour, the MM of P(3HB) was evaluated in cultures of *A. chroococcum* 7B using sucrose (2% w/w) supplemented with sodium acetate at different concentrations (from 2 to 5 g l^−1^). Under such conditions, the MM of P(3HB) decreased as the acetate concentration increased. These results, provided an original method for production of P(3HB) with predetermined MM within a wide range, from 2.7 × 10^2^ kDa (using 2% sucrose w/v and acetate 5 g l^−1^) to 1.5 × 10^3^ kDa (with sucrose as a sole carbon source).

#### Influence of pH

The pH of the broth culture is a critical parameter for the optimal production of P(3HB). Reports have been published about the influence of this parameter on the concentration and molecular weight of this polymer (Kusaka *et al*., 1998; Myshkina *et al*., 2008; Bocanegra *et al*., 2013).

In this line, Myshkina and colleagues (2008) reported in shake flask cultures using *A. chroococcum* strain 7B that the mean molecular weight (MMW) of P(3HB) was influenced by the pH of the broth culture, finding that the MMW was maximum (1485 kDa) when the bacterium was grown at neutral pH (7.0). A variation of pH in the interval of 6.0 to 8.0 allowed the synthesis of PHB of predetermined MMW in a wide range from 354 to 1485 kDa, determined by capillary viscometry.

On the other hand, Kusaka and colleagues (1998) reported that in cultures of recombinant *E. coli* XL-1 Blue (pSYL105), harbouring *C. necator* P(3HB) biosynthesis *phbCAB* genes, the MM of P(3HB) could be manipulated by changes in pH, reaching one of the highest values of MM reported for P(3HB) (11 × 10^3^ kDa) when *E. coli* cultures were grown at pH 6.5, and this value dropped up to 10-fold times (1.1 × 10^3^ kDa) when pH increased to 7.0.

More recently, Bocanegra and colleagues (2013) evaluated P(3HB) production by recombinant *E. coli* XL-1 Blue harbouring plasmid pSK::*phbCAB* at three different pHs (6.0, 6.5 and 7.0). Cultures in bioreactor using glucose as the sole carbon source at variable pH values (6.0, 6.5, or 7.0) allowed the production of P(3HB) with MMW varying between 2.0 and 2.5 × 10^3^ kDa. These values were significantly higher than those obtained by natural bacterial strains (0.5–1.0 MDa). However, in contrast to that reported by Kusaka *et al*., 1998, no influence of pH was observed on the MMW of the polymer produced (Table 5).

#### Influence of aeration conditions

There are reports in the literature where the influence of the aeration conditions on the MMW of P(3HB) has been evaluated. Quagliano and Miyazaki (1997) evaluated different levels of aeration in a stirred bioreactor for *A. chroococcum* 6B. These authors reported that at lower aeration (0.5 vvm), the MM of P(3HB) (determined by the intrinsic viscosity) was of 1.1 × 10^3^ kDa. In contrast, at higher aeration (2.5 vvm), the molecular weight significantly decreased at values of 1.0 × 10^2^ kDa. In addition, Myshkina and colleagues (2008) found that by culturing *A. chroococcum* 7B in shake flasks, the molecular mass of P(3HB) increased from 1.48 × 10^3^ to 1.67 × 10^3^ kDa when the agitation rate decreased from 250 to 190 r.p.m. (Table 5). However, the yield of P(3HB) on biomass was very similar in both conditions evaluated.

Previous studies in our group revealed that the MM of P(3HB) is strongly influenced by both the aeration condition and the strain tested (Peña *et al*., 2014). In that study, a maximal MM of 2.02 × 10^3^ kDa was observed for the P(3HB) isolated from the cultures of OPN mutant under low aeration conditions at 60 h of cultivation. A similar behaviour was observed in the polymer produced by the OP strain, obtaining a P(3HB) with a MW of 1.65 × 10^3^ kDa at the same time. In contrast, in the cultures at high aeration, the molecular weight of P(3HB) decreased to 1.01 × 10^3^ kDa and 5.51 × 10^2^ kDa for the OPN and parental strain (OP) respectively (Table 5).

Finally, it is important to point out that the MM can be controlled to some extent by genetic manipulation. An interesting example was reported by Hiroe and colleagues (2012). They showed that the concentration of active PHA synthase, relative to that of the enzymes supplying the monomer has a negative correlation with the P(3HB) molecular weight. They were able to construct strains producing a high molecular weight polymer by changing the order of the *phaA, phaB* and *phaC* genes within the operon, which in turn determines their relative expression level. Another example illustrating the effect of genetic changes on P(3HB) MM control was reported by Zheng and colleagues (2006). A deletion of 78 amino acid residues from the highly variable N-terminal fragment of the P(3HB) synthase of *C. necator*, resulted in a 60-fold increase in the average molecular weight, reaching a size of 2.84 × 10^3^ kDa. An α-helix structure was predicted in this region, and mutations disrupting this structure at amino acids 75 and 81 were shown to also increase 50-fold the size of the polymer, allowing simultaneously a higher production of the P(3HB).

## Conclusions and future prospects

In this article, several aspects about P(3HB) polymer production using different microorganisms and fermentation strategies have been reviewed. It is clear that the commercial applications of P(3HB) depend on the characteristics of the polymer. In this sense, it has been shown that the strain and culture conditions employed determine the molecular mass of the P(3HB) produced, and that this characteristic can also be further modified by genetic alteration of the producer strain. The understanding of the regulatory mechanisms controlling the synthesis of P(3HB) has also helped in some cases to construct mutants improved for P(3HB) production. In addition, some recombinant strains have shown to produce sufficient P(3HB) for large-scale production. The development of fermentation strategies has also shown promising results in terms of improving the productivity. Undoubtedly, the fed-batch fermentation and the multistage systems seem to be the more suitable strategies for improving the P(3HB) production. By using this kind of systems, it has been possible to reach a very high yields and productivities of P(3HB). Overall, the use of recombinant strains, in combination with a multistage fermentation process and raw materials for low cost could be a feasible strategy to optimize the P(3HB) production at the industrial level. However, the cost of the substrates for P(3HB) production and extraction of these materials is still the bottleneck, which limits the possibility to market them at larger scale. For this reason, the implementation of systems of production by mixed microbial cultures and wastes as substrates seems to have many advantages in the close future. In addition, the use of Archaeabacteria could be a feasible strategy to the PHA production, because they can utilize cheap carbon sources and are able to grow under extreme conditions, in which other microorganisms do not survive.
